# The relationship between Hill-Sachs lesion and scapular morphology in anterior shoulder instability

**DOI:** 10.1007/s00256-026-05268-2

**Published:** 2026-05-29

**Authors:** Haluk Yaka, Betül Digilli Ayaş, Meltem Oruç, Emine Aynur Çiçekcibaşi, Ali Adem, Baran Sarikaya, Mustafa Özer, Ulunay Kanatli

**Affiliations:** 1 Department of Orthopaedics & Traumatology, Necmettin Erbakan University School of Medicine, Konya, Turkey; 2 Department of Anatomy, Necmettin Erbakan University School of Medicine, Konya, Turkey; 3 Department of Radiology, Karaman State Hospital, Karaman, Turkey; 4 Department of Orthopaedics & Traumatology, Bilkent City Hospital, Ankara, Turkey; 5 Department of Orthopaedics & Traumatology, Gazi University School of Medicine, Ankara, Turkey

**Keywords:** Hill-Sachs lesion, Anterior shoulder instability, Scapular morphology, Critical shoulder angle, Glenoid inclination

## Abstract

**Objective:**

This study investigated the relationship between scapular morphological parameters (critical shoulder angle, glenoid inclination, and glenoid version) and Hill-Sachs lesion presence and size in anterior shoulder instability patients without critical glenoid bone loss (< 15%), using 3D computed tomography.

**Materials and methods:**

Computed tomography (CT) images and arthroscopic records of 93 patients without critical glenoid bone loss who had anterior shoulder instability surgery were reviewed. 3D CT reconstructions were made using 3D Slicer software. Critical shoulder angle (CSA), glenoid inclination (GI), and glenoid version (GV) were measured, and their associations with HSL length (HSL-L), HSL width (HSL-W), HSL depth (HSL-D) and HSL area (HSL-A) were analyzed. Patients were grouped by HSL presence, and statistical analyses were conducted.

**Results:**

HSLs were identified in 61 of 93 patients (32 HSL-negative). CSA was significantly higher in HSL-positive than HSL-negative patients (34.5 ± 2.9° vs 30.4 ± 1.7°, *P* < 0.001). Logistic regression showed CSA was independently associated with HSL presence (*P* = 0.002; OR = 2.7). CSA above 32.1° showed 77.8% sensitivity and 83.7% specificity for HSL presence. CSA correlated with HSL-L and HSL-A, and GI positively correlated with HSL size parameters (HSL-L, HSL-W, HSL-D and HSL-A). HSL presence and size were independent of dislocation number, age, sex, and side.

**Conclusions:**

HSL presence and size were associated with scapular morphology, with CSA independently predicting HSL presence at values above 32.1°. These findings apply to patients with anterior shoulder instability without critical glenoid bone loss (< 15%). CSA may serve as an ancillary parameter in stabilization surgery planning, particularly when choosing between Bankart repair and Bankart + remplissage.

## Introduction

Most traumatic anterior shoulder dislocations are accompanied by a Hill-Sachs lesion (HSL), which is defined as a compression fracture of the posterosuperior aspect of the humeral head resulting from impaction against the anterior glenoid rim [1]. Due to its complex pathomechanics and anatomical characteristics, the presence and extent of an HSL play an important role in anterior shoulder instability by influencing preoperative planning and postoperative outcomes [1–3]. It has been demonstrated that large and medially located HSLs accompanying isolated Bankart repair are associated with recurrent instability, often necessitating additional stabilization procedures beyond labral repair alone [4].

Previous studies have primarily focused on the relationship between glenoid bone defects and the size and location of HSLs. Peebles et al. reported a negative correlation between glenoid bone defect size and HSL dimensions [5]. In contrast, several other studies have demonstrated a positive correlation between the extent of glenoid bone loss and HSL size [6–8]. In addition to glenoid bone loss, glenoid morphology has also been implicated in HSL development. Wu et al. reported that a deeper and more concave glenoid configuration was associated with larger HSLs in patients with anterior shoulder instability [1].

Although scapular morphological parameters such as critical shoulder angle (CSA), glenoid inclination (GI), and glenoid version (GV) have been shown to influence shoulder biomechanics and various shoulder pathologies, their relationship with the presence and size of HSL has not been clearly established. Özer et al. demonstrated that increased CSA and GI were associated with greater tuberosity fractures in traumatic anterior shoulder dislocations, proposing that the increased superior deltoid force vector caused by high CSA may strengthen the attachment of the supraspinatus and infraspinatus to the greater tuberosity, thereby increasing posterosuperior traction forces and contributing to specific osseous injury patterns [9]. By analogy, a similar biomechanical mechanism may underlie HSL formation—high CSA may amplify the compressive force between the humeral head and the anterior glenoid rim at the moment of dislocation, potentially contributing to both the presence and size of HSL. However, this relationship has not been directly investigated.

Three-dimensional CT reconstructions were preferred over 2D imaging, as they provide more accurate and reproducible measurements of scapular morphological parameters by eliminating the influence of scapular rotation and patient positioning. Therefore, the aim of this study was to investigate the relationship between scapular morphology parameters, including CSA, GI, and GV, and the presence and size of Hill-Sachs lesions using three-dimensional computed tomography.

## Materials and methods

### Patient cohort

After obtaining institutional ethical approval, data from all consecutive patients who underwent arthroscopic treatment for anterior shoulder instability by a single senior surgeon in a tertiary hospital between January 2020 and January 2023 were retrospectively analyzed. Of the 122 eligible patients, 93 met the inclusion criteria and were included in the final analysis. Inclusion criteria were as follows: patients who underwent arthroscopic treatment for anterior shoulder instability with at least one clinically documented anterior glenohumeral dislocation, available preoperative CT imaging, available arthroscopic records and operative notes, and age between 18 and 45 years with glenoid bone loss less than 15%. Anterior shoulder instability was defined as at least one clinically documented anterior glenohumeral dislocation requiring medical evaluation and/or reduction, supported by consistent physical examination findings and preoperative imaging. When available, the index dislocation was confirmed using emergency department records and/or radiographs obtained at the time of injury. Exclusion criteria included absence of CT imaging; glenoid bone loss > 15%; shoulder arthrosis; concomitant glenoid or humeral fractures; age < 18 or > 45 years; rotator cuff rupture; congenital deformities; neurological disease; previous upper extremity surgery or fracture; multidirectional instability; generalized ligamentous laxity; and inadequate imaging quality. Imaging was considered inappropriate when image quality was insufficient for reliable three-dimensional reconstruction and measurement due to motion artifacts, incomplete visualization of the scapula or humeral head, or inadequate field of view. The age range was restricted to minimize the potential confounding effects of age-related degenerative changes. Patients with glenoid bone loss exceeding 15% were excluded because significant glenoid bone loss is known to independently influence both the presence and morphology of Hill-Sachs lesions, which would have introduced a major confounding variable and potentially masked the independent contribution of scapular morphological parameters to HSL formation [10].

### Imaging and image analysis

All images were examined using a dual-source 256-multidetector CT Somatom Drive (Siemens Healthineers, Germany) with acquisition parameters of 80 and 140 kV, 89 and 178 mA, a rotation time of 0.28 s, collimation of 256 × 0.625, and a field of view (FOV) of 220 mm. CT images of the patients were saved in Digital Imaging and Communications in Medicine (DICOM) format and uploaded to 3D Slicer (http://www.slicer.org), an open-source software platform [11]. After the CT images were displayed in 3D Slicer as scrollable in three planes (sagittal, coronal, and axial), a new segment was created using the segment-editor module of the software. Three-dimensional models of the scapula and humerus bones were generated using a threshold tool. Length, angle, and area measurements were taken from three-dimensional bone models using the Markings tool within the software. All measurements were independently performed by two observers: an orthopedic surgeon with 7 years of experience in shoulder surgery (Observer 1) and an anatomist with 5 years of experience in musculoskeletal research (Observer 2). Both observers were blinded to each other's measurements and performed all measurements in two separate sessions with a minimum 4-week interval between sessions. In addition, a radiologist with 6 years of experience in musculoskeletal imaging reviewed all imaging data and the 3D reconstructions, and checked the measurements performed by the two observers for accuracy and consistency. The radiologist did not independently perform measurements. Each observer performed measurements twice with a 4-week interval, resulting in four sets of measurements per patient. Final values were calculated by averaging these four measurements. To ensure measurement accuracy and account for potential rotational variability of the 3D model, all measurements were performed by first orienting the scapula in the standard anteroposterior position, followed by dynamic multiplanar rotation of the 3D model to confirm the measurement from multiple viewing angles. This multiplanar confirmation approach represents a key advantage of 3D CT over conventional 2D measurements, as it allows observers to verify the accuracy of each measurement by examining the anatomical landmarks from various orientations rather than relying on a single fixed projection. The same methodology was applied consistently for all scapular morphological parameters including CSA, GI, and GV.

**Fig. 1:**
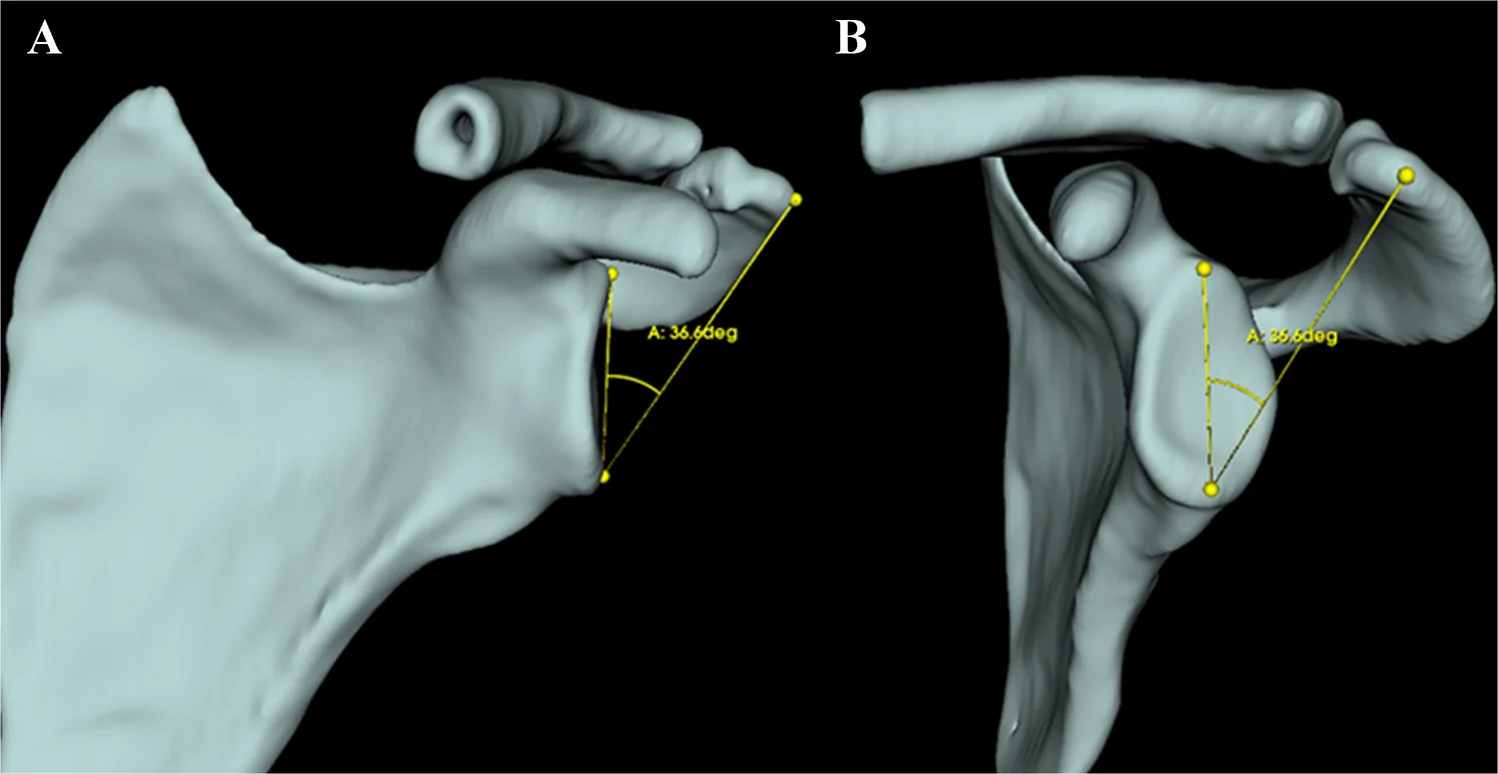
Demonstration of the measurement of critical shoulder angle (CSA) on three-dimensional computed tomography (3D-CT). **A** Anterior view of CSA measurement. **B** Lateral view of CSA measurement

The critical shoulder angle (CSA) is defined as the angle between a line joining the superior and inferior bony edges of the glenoid fossa and a line joining the inferior edge of the glenoid fossa and the lowest lateral aspect of the acromion [12–14]. It has been shown that CSA measurement on three-dimensional computed tomography can be an alternative to measurements made on radiography (Fig. 1) [12].

**Fig. 2:**
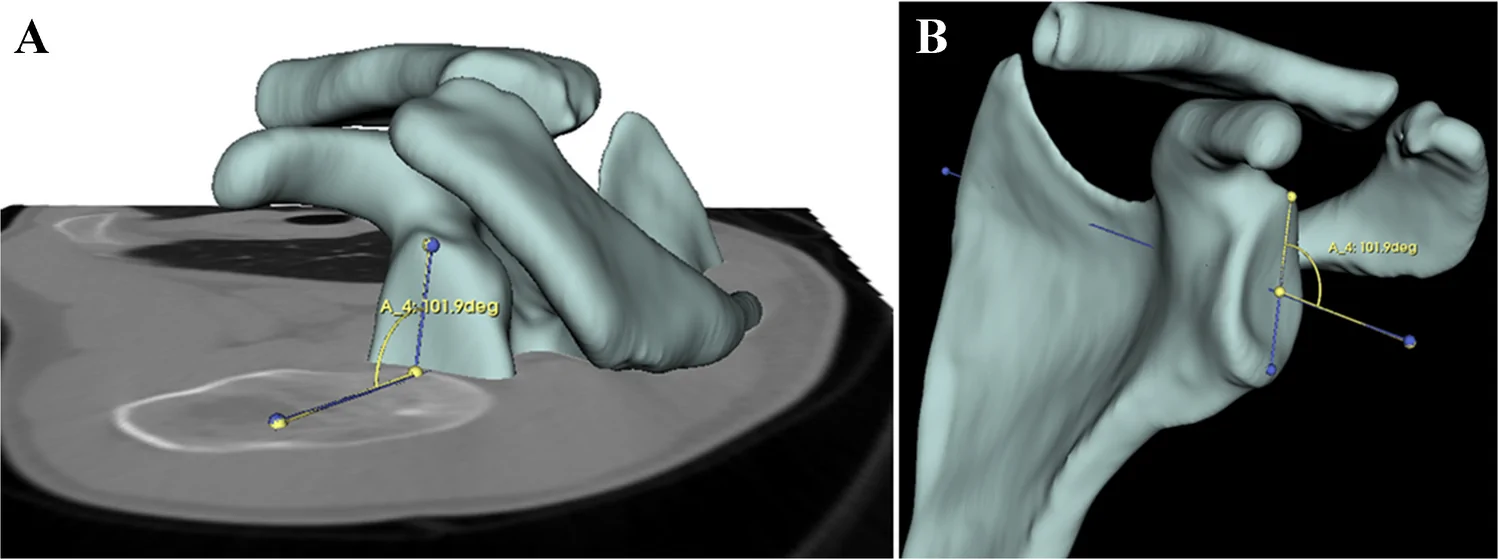
Demonstration of glenoid inclination (GI) measurement on 3D-CT. **A** View from lateral. **B** View from anterior

Glenoid inclination (GI) is the angle between the supraspinatus fossa and the glenoid articular surface [15, 16]. 3D-CT studies have shown that GI measurement is more accurate than 2D-CT measurements (Fig. 2) [17].

**Fig. 3:**
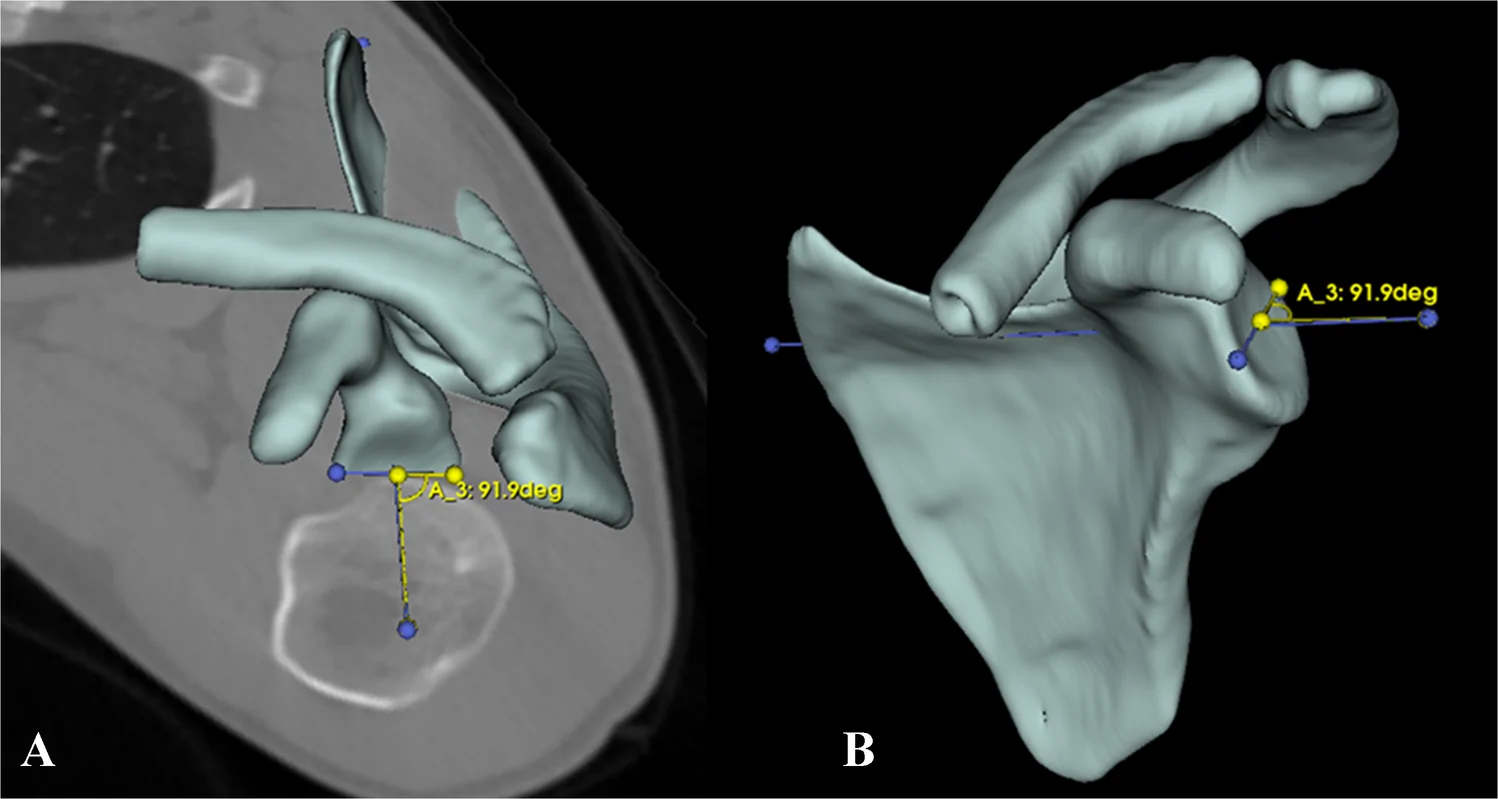
View of the glenoid version (GV) measurement on 3D-CT. **A** View from superior. **B** View from anterior

Glenoid version (GV) is the angle between the tangent of the line extending from the medial scapula to the middle of the glenoid articular surface and the glenoid articular surface in the axial plane [18]. In 3D-CT imaging, transverse scapular plane and coronal scapular planes were determined and GV measurements were made (Fig. 3) [17].

For CSA, the scapula was first oriented in the standard anteroposterior position, with multiplanar confirmation to identify the superior and inferior bony edges of the glenoid fossa and the lowest lateral aspect of the acromion. For GI, the supraspinatus fossa and glenoid articular surface were identified in the coronal scapular plane. For GV, the transverse and coronal scapular planes were determined to measure the angle between the tangent of the line extending from the medial scapula to the middle of the glenoid articular surface. All orientations were confirmed from multiple viewing angles to minimize measurement error.

**Fig. 4:**
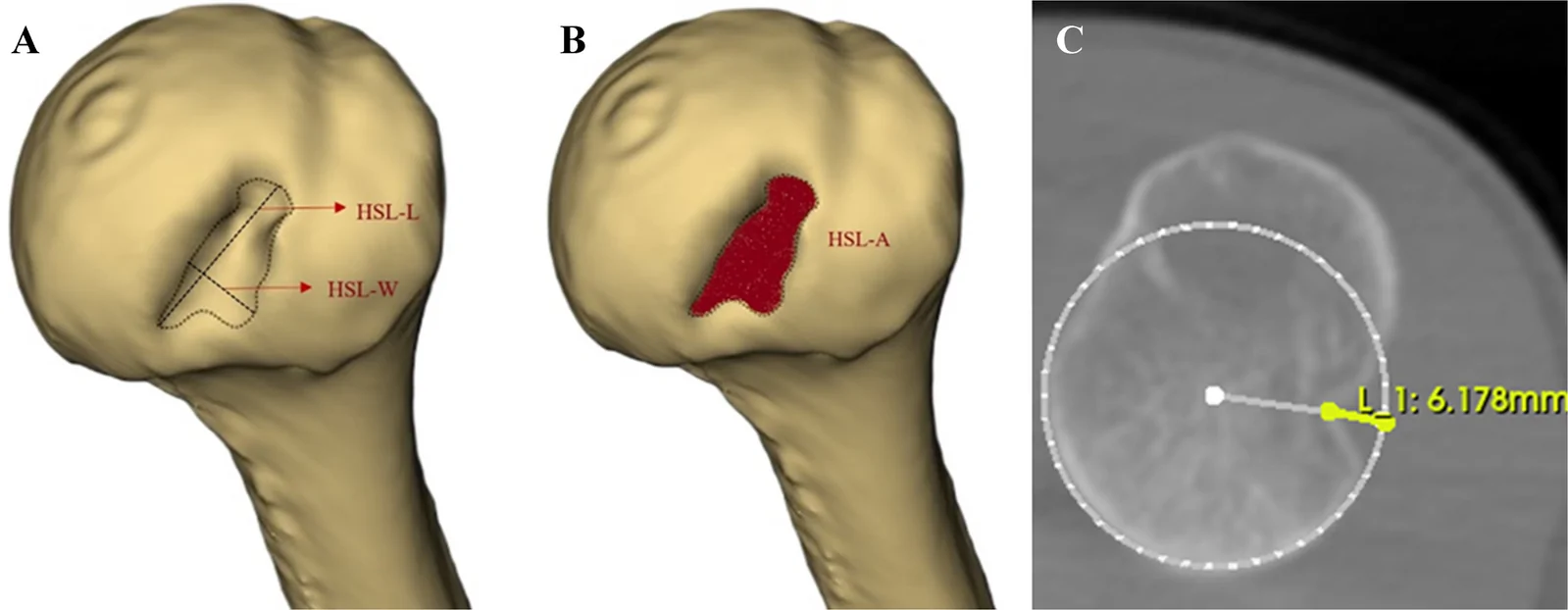
Representation of Hill-Sachs lesion (HSL)-related measurements on 3D-CT. **A** HSL length (HSL-L) and HSL width (HSL-W) measurement. **B** HSL area (HSL-A) measurement. **C** HSL depth (HSL-D) measurement

HSL was measured as described by Ozaki et al. [19]. In 3D CT, the length of the major axis of the HSL was measured as the length of the HSL (HSL-L), and the length of the minor axis was measured as the width of the HSL (HSL-W) (Fig. 4). On axial CT images obtained perpendicular to the longitudinal axis of the humeral shaft, a virtual circle including the articular surface of the humeral head was drawn, and the longest length between the base of the Hill-Sachs lesion and the corresponding arc was measured as the depth of the HSL (HSL-D) (Fig. 4). In the axial section with the largest circle, the diameter of the circle was measured as the diameter of the humeral head. In addition to these defined measurements, the borders of the HSL were marked and the area between the points was measured as the HSL area (HSL-A). The absence of Hill-Sachs lesions was confirmed using multiplanar axial and three-dimensional CT reconstructions (Fig. 4). In patients in whom no lesion was identified on CT, arthroscopic images were additionally reviewed to corroborate the absence of a humeral head impaction defect. Patients were classified as HSL-negative when no evidence of a humeral head impaction defect was identified on arthroscopic evaluation, axial CT, or three-dimensional CT reconstructions.

### Statistical analysis

Intrarater and interrater reliability analyses were checked by intraclass correlation coefficient (ICC). Data analysis was performed using SPSS software (IBM-SPSS 22.0, Armonk, NY, USA). After ICC analysis, mean values, standard deviations were calculated by averaging the four measurements. Post-hoc power calculations were carried out using the G*Power software (version 3.1.9.4, Heinrich Heine University, Düsseldorf) with an alpha error of 0.05 and a two-tailed significance level. Skewness of the data was checked by Shapiro–Wilk test. The t-test was used for the comparison of parametric independent variables and the Mann–Whitney U test was used for the comparison of nonparametric independent variables. Pearson and Spearman-Rho tests were used for correlation analyses. Receiver operating characteristic (ROC) analysis was performed to explore a potential threshold value of CSA associated with the presence of Hill-Sachs lesions in this cohort. Calculations with *p* < 0.05 were considered significant. Logistic regression analysis was performed to determine whether the parameters were independently associated with the presence of Hill-Sachs lesions.

## Results

Of the 93 patients, 61 (65.6%) (53 males/8 females) had HSL, while 32 (34.4%) (27 males/5 females) had no bone defect (HSL) on both arthroscopic images, axial CT and 3D CT imaging. The mean age of patients with HSL was 27.3 ± 7.5 years (18–45), while the mean age of patients without HSL was 28.8 ± 8.5 years (18–45) and there was no significant difference between them (*P* = 0.652). There was no significant difference between the patient groups in terms of gender and side (*P* = 0.236, *P* = 0.563 respectively). The mean number of dislocations in patients with HSL was 5.6 ± 5 (1–14), while the mean number of dislocations in patients without HSL was 3.7 ± 1.6 (1–5) and there was no significant difference between them (*P* = 0.089). Of the 61 patients with HSL, 17 (27.9%) were first dislocations and 44 (72.1%) were recurrent dislocations. Of the 32 patients without HSL, 6 (18.8%) were first dislocations and 26 (81.2%) were recurrent dislocations, and there was no significant relationship between the presence of HSL and first or recurrent dislocation (*P* = 0.481).HSL was present in 17 (73.9%) of 23 patients with initial dislocation.Of the 70 patients with recurrent dislocation, 44 (62.9%) had HSL (demographic information is summarized in Table 1).

**Table 1:** Demographic information of patients (values are presented as *n* (% within group))

	**HSL(+) group**	**HSL(−) group**	***P* value**
Age (years ± SD) (min–max)	27.3 ± 7.5 (18–45)	28.8 ± 8.5 (18–45)	0.652
Gender (M/F)	53/8 (86.9%/13.1%)	27/5 (84.4%/15.6%)	0.236
Side (R/L)	42/19 (68.9%/31.1%)	25/7 (78.1%/21.9%)	0.563
Dislocation number	5.6 ± ´5 (1–14)	3.7 ± 1.6 (1–5)	0.089
First dislocation (*n*)	17 (27.9%)	6 (18.8%)	0.481
Recurrent dislocation	44 (72.1%)	26 (81.2%)	

The mean CSA value was 34.5º ± 2.9º (29.6º−42.5º) in patients with HSL and 30.4º ± 1.7º (28º−34.4º) in patients without HSL and CSA was significantly higher in patients with HSL (*P* < 0.001). The mean GI value was 9.7º ± 4.5º (3.9–17.2º) in patients with HSL and 8.4º ± 5.2º (−3.4º−13.6º)in patients without HSL, and there was no significant relationship between patients with and without HSL in terms of GI (*P* = 0.475). The mean GV value was 1.1º ± 5º (−7.1—9.3º) in patients with HSL and 0.8º ± 2.5º (−5.4º−6.2º) in patients without HSL, and there was no significant relationship in terms of GV between patients with and without HSL (*P* = 0.749). The mean humeral head radius was 24.1 ± 1.1 mm (23–27) in patients with HSL and 22.7 ± 1.2 mm (22–25) in patients without HSL, and there was no significant relationship between patients with and without HSL in terms of humeral head radius (*P* = 0.763) (Table 2).

**Table 2:** Comparison of the groups in terms of radiologic measurements (*CSA* critical shoulder angle, *GI* glenoid inclination, *GV* glenoid version, *HSL-L* Hill-Sachs lesion length, *HSL-W* Hill-Sachs lesion width, *HSL-D* Hill-Sachs lesion depth, *HSL-A* Hill-Sachs lesion area)

	HSL(+) group	HSL(−) group	*P *value
CSA (mean ± SD) (min–max)	34.5° ± 2.9° (29.6–42.5°)	30.4° ± 1.7° (28–34.4°)	< 0.001*
GI (mean ± SD) (min–max)	9.7° ± 4.5° (3.9–17.2°)	8.4° ± 5.2° (− 3.4–13.6°)	0.475
GV (mean ± SD) (min–max)	1.1° ± 5° (− 7.1–9.3°)	0.8° ± 2.5° (− 5.4–6.2°)	0.749
HSL-L (mean ± SD) (min–max)	18.5 ± 10.6 mm (6.6–38.4)		
HSL-W (mean ± SD) (min–max)	7.7 ± 4.7 mm (4.1–19.7)		
HSL-D (mean ± SD) (min–max)	7.1 ± 4.5 mm (2.3–15.8)		
HSL-A (mean ± SD) (min–max)	1.8 ± 2.2 cm^2^ (0.25–6.93)		

*Statistically significant, *P* < 0.05

**Fig. 5:**
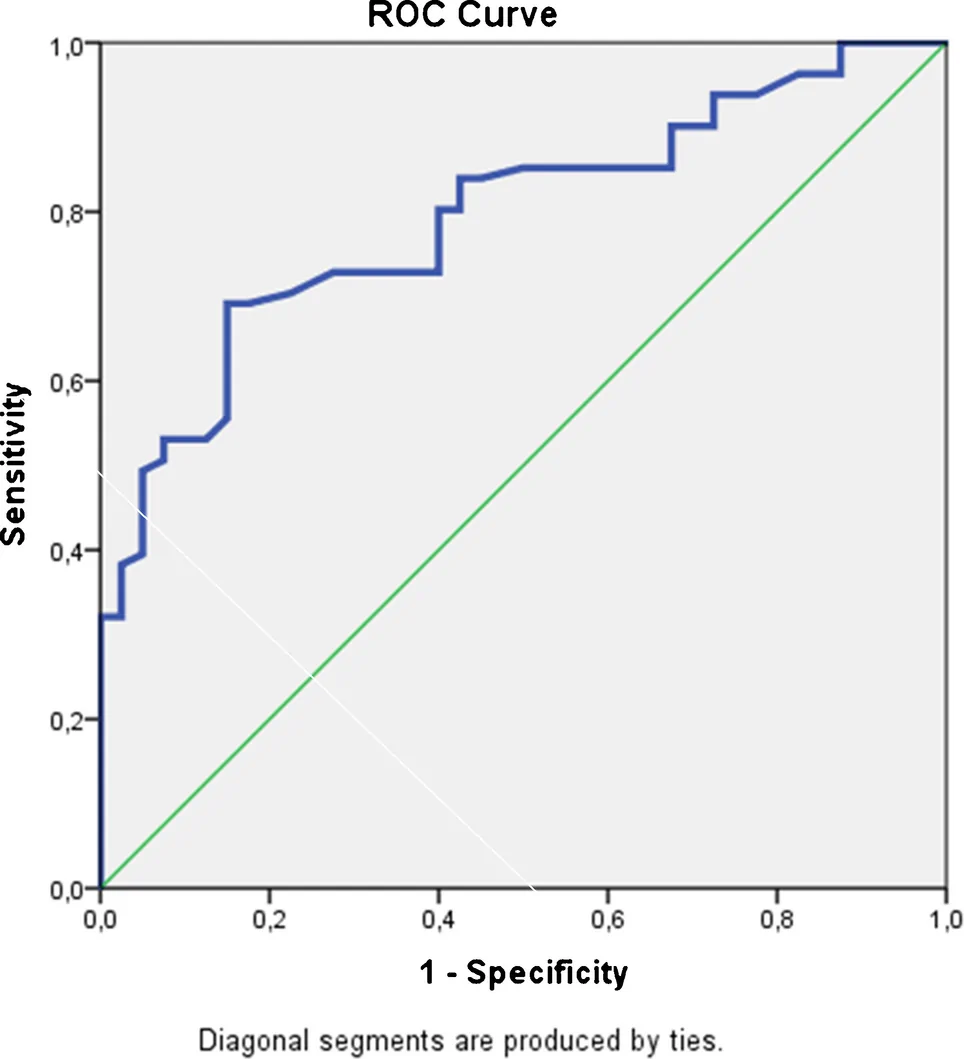
ROC curve of critical shoulder angle (CSA) associated with the presence of Hill-Sachs lesion (HSL) (AUC (area under the curve) 0.892, 95% CI of AUC 0.809–0.964)

Logistic regression analysis with CSA, GI, GV, gender, side, and number of dislocations showed that CSA was independently associated with the presence of HSL (*P* = 0.002, OR: 2.7).CSA values above 32.1º were associated with the presence of HSL, showing 77.8% sensitivity and 83.7% specificity in this cohort (Fig. 5) (AUC: 0.892, 95%CI of AUC: 0.809–0.964).

The mean HSL-Lwas 18.5 ± 10.6 mm (6.6–38.4), HSL-W 7.7 ± 4.7 mm (4.1–19.7), HSL-D 7.1 ± 4.5 mm (2.3–15.8), and HSL-A 1.8 ± 2.2 cm^2^ (0.25–6.93) (Table 2). When parameters related to HSL measurements were compared with scapula-related parameters, CSA was significantly positively correlated with HSL-L and HSL-A (*P* = 0.015, r = 0.551; *P* = 0.024, r = 0.514 respectively). GI was significantly positively correlated with HSL-L, HSL-W, HSL-D and HSL-A (*P* = 0.022, r = 0.521; *P* = 0.002, r = 0.656; *P* = 0.029, r = 0.501; *P* = 0.007, r = 0.597 respectively). No significant correlation was observed between GV and HSL parameters (Table 3).

**Table 3:** Correlation relationships between scapula-related parameters and HSL size–related parameters (*r*, correlation coefficient; *statistically significant, *P* < 0.05)

	CSA	GI	GV
HSL-L	*P* = 0.015*, *r* = 0.551	*P* = 0.022*, *r* = 0.521	*P* > 0.05
HSL-W	*P* > 0.05	*P* = 0.002*, *r* = 0.656	*P* > 0.05
HSL-D	*P* > 0.05	*P* = 0.029*, *r* = 0.501	*P* > 0.05
HSL-A	*P* = 0.024*, *r* = 0.514	P = 0.007*, *r* = 0.597	*P* > 0.05

The results of interrater and intrarater reliability analyses were between 0.79 and 0.95, indicating good and excellent reliability (observer-1:*****, observer-2: *****) (Table 4).

**Table 4:** ICC results for both groups (*CSA* critical shoulder angle, *GV* glenoid version, *GI* glenoid inclination, *HSL-L* Hill-Sachs lesion length, *HSL-W* Hill-Sachs lesion width, *HSL-D* Hill-Sachs lesion depth, *HSL-A* Hill-Sachs lesion area) (HSL-related measurements were not applicable in the HSL-negative group due to the absence of a measurable lesion)

Radiological parameter	HSL(+) group	HSL(−) group
CSA (intra-rater observer-1/intra-rater observer-2/inter-rater)	0.87/0.83/0.84	0.86/0.82/0.85
GV (intra-rater observer-1/intra-rater observer-2/inter-rater)	0.89/0.86/0.87	0.88/0.84/0.85
GI (intra-rater observer-1/intra-rater observer-2/inter-rater)	0.85/0.79/0.82	0.83/0.80/0.82
HSL-L (intra-rater observer-1/intra-rater observer-2/inter-rater)	0.95/0.90/0.91	
HSL-W (intra-rater observer-1/intra-rater observer-2/inter-rater)	0.92/0.90/0.90	
HSL-D (intra-rater observer-1/intra-rater observer-2/inter-rater)	0.88/0.81/0.86	
HSL-A (intra-rater observer-1/intra-rater observer-2/inter-rater)	0.85/0.80/0.82	

## Discussion

One of the most important findings of this study was that there was an association between the presence of HSL and increased CSA. CSA was associated with HSL independently of other parameters and CSA values above 32.1º showed 77.8% sensitivity and 83.7% specificity for the presence of HSL in this cohort. The second important finding of the study was that CSA was significantly correlated with HSL-L and HSL-A and GI was significantly correlated with HSL-L, HSL-W, HSL-D and HSL-A, suggesting a morphological association between HSL size and both acromial morphology and glenoid inclination, which are closely related to shoulder biomechanics. However, given the multifactorial nature of HSL formation, these findings should be interpreted as exploratory rather than confirmatory, and causality cannot be inferred from this association. Although repeated instability episodes are traditionally considered a contributing factor in the development of Hill-Sachs lesions, the findings of the present study did not demonstrate a statistically significant association between dislocation number and HSL presence. However, patients with HSL demonstrated higher mean dislocation numbers compared with those without HSL, suggesting a positive trend toward higher dislocation numbers in the HSL-positive group (P = 0.089) that did not reach statistical significance in this cohort. This trend may become more apparent in larger study populations or in cohorts with different instability characteristics. These findings indicate that the occurrence of Hill-Sachs lesions may not be solely dependent on the frequency of dislocation events, but rather influenced by underlying shoulder biomechanics, as reflected by scapular morphology parameters such as the critical shoulder angle.

Kim et al. found the incidence of HSL to be 58% after initial dislocations in a study on magnetic resonance imaging [20]. Yiannakopoulos et al. found a 65% incidence of HSL in arthroscopic imaging [21]. Calandra et al. also found HSL in only 47% of cases on arthroscopic imaging [22]. In their pilot study, Ruiz Ibán et al. compared HSL dimensions before and after reduction in 20 patients with first-time dislocation. They showed that HSL was present in all cases and the HSL size increase was over 25% in three quarters of the cases. [23]. Owens et al. and also Taylor and Anciero reported a similarly high prevalence of HSL. [24, 25] In our study, HSL was present in 73.9% of first dislocation cases. When these results obtained in the literature and our results are evaluated, it seems that the incidence of HSL after first dislocation will continue to be variable. One of the interesting findings of our study was that HSL was present in 73.9% of patients with initial dislocation, while 62.9% of those with recurrent dislocations had HSL. Although the majority of studies report a higher prevalence of HSL in recurrent dislocations compared to first-time dislocations, Horst et al. demonstrated that recurrence of shoulder dislocation was not associated with a higher number of Hill-Sachs lesions, nor did defect sizes differ significantly between first-time and recurrent dislocation groups [26]. In light of this, our finding may suggest that in a subset of patients, HSL formation may be more strongly influenced by biomechanical factors such as scapular morphology—particularly CSA—rather than dislocation frequency alone. Patients without HSL after initial dislocation may represent a biomechanically distinct group in whom the forces generated during dislocation are insufficient to produce an impaction defect, potentially making them less likely to develop HSL in subsequent events. However, this remains speculative and requires confirmation in larger prospective studies.

It is known that the most common lesion accompanying shoulder dislocations is probably the HSL, but the factors determining the size of the HSL continue to be debated; it is known that the upward forces causing shoulder dislocation and ligament hyperlaxity are effective on the HSL [23, 26]. Previous studies have shown that in cases with ligament hyperlaxity, the force required for shoulder dislocation will be smaller, which may explain the smaller size of the HSL with lower shear and pressure effect after dislocation [26–28]. It has been reported in previous studies that HSL is less likely to occur in recurrent dislocations with lower shear and compressive forces, and this information may support the fact that the incidence of HSL in recurrent dislocations in our study was lower than the prevalence of HSL in initial dislocations [26]. Thus, the fact that dislocation occurs with lower shear and compressive forces in patients without HSL suggests that the initial dislocation can easily turn into a recurrent dislocation. In patients with normal ligamentous laxity, the effect of more force is responsible for the occurrence of HSL [26]. In the light of this information in the literature, it can be thought that the compressive force between the humeral head and the glenoid after shoulder dislocation may also be related to CSA, which is closely related to shoulder biomechanics. It is known that a high CSA causes the superior vector of the force exerted by the deltoid to be greater in an anatomical shoulder, and the fact that the force exerted by the deltoid on the humerus after dislocation is more superolateral may explain why a high CSA is associated with both the presence and size of HSL [9, 13]. In addition, a glenoid with a higher superior inclination may result in a more pointed/sharp anteroinferior glenoid rim, which may cause more compressive force and be associated with a higher HSL size. Wu et al. found that HSL dimensions were greater in cases with greater natural depth of the glenoid and this may be due to a more concave glenoid associated with a more pointed/sharp anteroinferior glenoid rim [1]. In this respect, the two studies support each other.

The presence and extent of HSL is very likely related to multiple factors, such as ligament laxity, the reduction maneuver, or the time between initial dislocation and reduction as shown by Ruiz Ibán et al. [23]. In our study, in the analysis performed with the number of dislocations, age, gender, side and radiologic measurement parameters, CSA was associated with HSL independently of other factors, but showed 77.8% sensitivity and 83.7% specificity, indicating that many factors that we did not evaluate in our study are associated with HSL.

Özer et al. compared patients with isolated anterior shoulder instability (ASI) to those with ASI accompanied by greater tuberosity fracture, and demonstrated that greater tuberosity fracture accompanied ASI with 88.2% sensitivity and 88.9% specificity in patients with CSA above 38º, and with 70.6% sensitivity and 72.2% specificity in patients with GI above 14.5º [9]. They explained the accompaniment of ASI with a greater tuberosity fracture as the increased deltoid superior vector caused by high CSA may provide a stronger attachment of the supraspinatus and infraspinatus muscles to the greater tuberosity and therefore the forces pulling the greater tuberosity posterosuperiorly may avulse [9]. In the same way, high CSA may have strengthened the attachment of the supraspinatus and infraspinatus to the greater tuberosity over time, pulling the greater tuberosity more strongly posterosuperiorly after shoulder dislocation, increasing the pressure between the humeral head and the glenoid, leading to the occurrence of HSL and a larger size of HSL. In our study, there seems to be a relationship between CSA above 32.1º and the presence of HSL, while in the study of Özer et al., CSA values above 38º seem to be associated with greater tuberosity fracture. From this point of view, HSL, which is called impression fracture, may occur at CSA values above 32.1º, while a greater tuberosity fracture, which is a complete fracture, may occur at CSA values above 38º, which indicates that the term impression fracture, which was previously used in the literature to describe HSL as an incomplete fracture of the posterosuperior humerus, may turn into a complete fracture as CSA increases [29, 30]. However, as in the study by Özer et al., this scenario remains a hypothesis and further studies are required. These findings reflect a morphologic association rather than a clinically actionable threshold and should not be interpreted as evidence of causality.

The findings of the present study have several potential clinical implications. From a radiological perspective, the identification of a high CSA on preoperative CT imaging may serve as an indicator of increased likelihood of HSL presence, prompting radiologists to perform a more thorough evaluation of the humeral head for impaction defects in these patients. This may be particularly relevant in cases where HSL is subtle or borderline on standard imaging sequences.

From a surgical planning perspective, CSA may serve as an ancillary morphological parameter when deciding between Bankart repair alone versus Bankart repair combined with remplissage, particularly in borderline cases with subcritical glenoid bone loss. In a randomized controlled study, MacDonald et al. examined the outcomes of patients with subcritical glenoid bone defects who underwent Bankart repair with and without remplissage [31]. They found recurrent dislocation rates of 4% and 18% respectively, demonstrating that remplissage significantly decreased recurrence regardless of HSL size. Woodmass et al. conducted a similar study and reported comparable results [32]. Based on the findings of the present study, patients with high CSA are more likely to have larger HSL, which may further support the addition of remplissage in these cases. Conversely, in patients with low CSA and therefore smaller expected HSL, Bankart repair alone may be sufficient. However, these implications remain hypothesis-generating and further prospective studies are needed to validate the role of CSA in surgical decision-making for anterior shoulder instability.

The first limitation of this study is the lack of information regarding the time between the initial dislocation and reduction and the reduction maneuver applied. In addition, body mass index, occupation of the patients and the mechanism of the initial dislocation were unknown. The small number of patients is a limitation because preoperative tomography was not performed in all patients in our clinic and those without tomography imaging were not included in the study. Furthermore, we acknowledge that the exclusion of patients with glenoid bone loss exceeding 15% limits the generalizability of our findings to patients with anterior shoulder instability without critical glenoid bone loss. In addition, the small number of patients and the statistical evaluation of a small number of patients by dividing them into subgroups increases the possibility of the results being randomized. Given the number of parameters analyzed, the observed associations should be interpreted with caution and considered exploratory rather than confirmatory. 

## Conclusion

HSL presence and size were associated with scapular morphology. CSA was independently associated with HSL presence, CSA values above 32.1º were associated with the presence of HSL in this cohort. These findings are applicable to patients with anterior shoulder instability without critical glenoid bone loss (glenoid bone loss < 15%). CSA may be considered as an ancillary morphologic parameter when planning stabilization surgery, particularly in borderline cases where Bankart repair versus Bankart + remplissage is debated.

## Data Availability

Data are available from the corresponding author upon reasonable request.
